# Supplementary material for: A geospatial analysis comparing wastewater-monitored sewershed and statewide populations for 32 states participating in CDC’s National Wastewater Surveillance System, 2021-2024
Source: PLOS Glob Public Health. 2026 Apr 22;6(4):e0006243. doi: 10.1371/journal.pgph.0006243 (PMC13102245; doi:10.1371/journal.pgph.0006243)
Supplement: Attachment — Submitted filename: PGPH-D-25-03983-rev.pdf [file pgph.0006243.s008.pdf]

PLOS Global Public Health

A geospatial analysis comparing wastewater-monitored sewershed and statewide populations for 32 states participating in CDC's National Wastewater Surveillance System, 2021-2024

--Manuscript Draft--

**Manuscript Number:**	PGPH-D-25-03983
**Article Type:**	Research Article
**Full Title:**	A geospatial analysis comparing wastewater-monitored sewershed and statewide populations for 32 states participating in CDC's National Wastewater Surveillance System, 2021-2024
**Short Title:**	Comparison of wastewater-monitored and statewide populations
**Corresponding Author:**	Stacie Reckling<br>North Carolina State University<br>Raleigh, North Carolina UNITED STATES OF AMERICA
**Order of Authors:**	Stacie Reckling
	Heather Reese, PhD
	Lisa P. Oakley, PhD
	Helena Mitasova, PhD
**Keywords:**	wastewater monitoring; wastewater surveillance; sewershed; geospatial; National Wastewater Surveillance System; demographics; social vulnerability; representativeness
**Abstract:**	Wastewater monitoring analyzes samples of untreated wastewater for pathogens to track community-level disease trends. A sewershed polygon helps define the sample population by depicting the community area that contributes sewage to the wastewater sample. This study utilized sewershed geospatial data submitted to the Centers for Disease Control and Prevention's National Wastewater Surveillance System (CDC NWSS) to determine the wastewater-monitored population characteristics and assess how well the wastewater-monitored population represents the broader population. In a geographic information system, we intersected sewershed, state, and county polygons with US Census data and social vulnerability data to calculate the proportion of the population with certain demographic and social vulnerability characteristics in each geographic unit. We compared the aggregated sewershed population within the state to the statewide population for wastewater monitoring sites sampling during the Fall of 2024. In some states, the wastewater-monitored population reflected the characteristics of the statewide population well. However, in other states, we observed that the wastewater-monitored population included a higher proportion of racial and ethnic minorities, a lower proportion of Whites, had higher educational attainment, lower median household income, or higher social vulnerability related to racial and ethnic minority status. In four states, Colorado, New York, North Carolina, and Wisconsin, we further assessed wastewater representativeness; we compared the distributions of sewershed populations with county populations and examined how representativeness has changed across eight time points during 2021-2024. We found that over time, differences between sewershed and statewide population characteristics were only slightly affected by changes in the number of sampling sites and population. State and local health departments, as well as CDC NWSS, can utilize information about the wastewater sample population and how it compares to the state population to strengthen the use of wastewater monitoring data for public health action.
**Additional Information:**	
**Question**	**Response**
**Financial Disclosure**	The authors received no financial support for the research, authorship, and/or publication of this article.

Powered by Editorial Manager® and ProduXion Manager® from Aries Systems Corporation

Enter a financial disclosure statement that describes the sources of funding for the work included in this submission and the role the funder(s) played. This includes grants and any commercial funding of the work or authors.<br><br>This statement will be typeset if the manuscript is accepted for publication.<br><br>*Please review the submission guidelines and the instructions link below for detailed requirements and guidance.*	
**Competing Interests**<br><br>On behalf of all authors, disclose any competing interests that could be perceived to bias this work.<br><br>This statement will be typeset if the manuscript is accepted for publication.<br><br>*Please review the instructions link below and PLOS Global Public Health's competing interests policy to determine what information must be disclosed at submission.*	The authors declare that they have no known competing financial interests or personal relationships that could have appeared to influence the work reported in this paper.
**Data Availability**<br><br>Before publication, Authors are required to make fully available and without restriction all data underlying their findings. Please see our PLOS Data Policy page for detailed information on this policy.<br><br>A **Data Availability Statement**, detailing where the data can be accessed, is required at first submission. Insert your Data Availability Statement in the box below.	Links to publicly available databases used in this project are provided in the reference section. The data supporting the findings of this study are available in the Supplemental Information. Sewershed polygons are unavailable to access due to concerns about sensitive infrastructure information.

Please see the data reporting section of our submission guidelines for instructions on what you need to include in your Data Availability Statement.<br><br>This statement will be typeset if the manuscript is accepted for publication.<br><br>*PLOS allows rare exemptions to address legal and ethical concerns. If you have legal or ethical restrictions, please use the box below to detail these in full sentences for the Journal team to consider.*	

Manuscript

Click here to access/download;Manuscript;nwss_population_representativene

A geospatial analysis comparing wastewater-monitored sewershed and statewide populations for 32 states participating in CDC's National Wastewater Surveillance System, 2021-2024

Stacie Reckling[1,2] *, Heather Reese[1], Lisa P. Oakley[1], and Helena Mitasova[2,3]

[1] Centers for Disease Control and Prevention, 1600 Clifton Road NE, Atlanta, GA 30333, United States of America

[2] Center for Geospatial Analytics, North Carolina State University, Raleigh, North Carolina, United States of America

[3] Marine Earth and Atmospheric Sciences, North Carolina State University, Raleigh, North Carolina, United States of America

* Corresponding author

Email: skreckli@ncsu.edu (SR)

Abstract

Wastewater monitoring analyzes samples of untreated wastewater for pathogens to track community-level disease trends. A sewershed polygon helps define the sample population by depicting the community area that contributes sewage to the wastewater sample. This study utilized sewershed geospatial data submitted to the Centers for Disease Control and Prevention's National Wastewater Surveillance System (CDC NWSS) to determine the wastewater-monitored population characteristics and assess how well the wastewater-monitored population represents the broader population. In a geographic information system, we intersected sewershed, state, and county polygons with US Census data and social vulnerability data to calculate the proportion of the population with certain demographic and social vulnerability characteristics in each geographic unit. We compared the aggregated sewershed population within the state to the statewide population for wastewater monitoring sites sampling during the Fall of 2024. In some states, the wastewater-monitored population reflected the characteristics of the statewide population well. However, in other states, we observed that the wastewater-monitored population included a higher proportion of racial and ethnic minorities, a lower proportion of Whites, had higher educational attainment, lower median household income, or higher social vulnerability related to racial and ethnic minority status. In four states, Colorado, New York, North Carolina, and Wisconsin, we further assessed wastewater representativeness; we compared the distributions of sewershed populations with county populations and examined how representativeness has changed across eight time points during 2021-2024. We found that over time, differences between sewershed and statewide population characteristics were only slightly affected by changes in the number of sampling sites and population. State and local health departments, as well as CDC NWSS, can utilize information about the wastewater sample population and how it compares to the state population to strengthen the use of wastewater monitoring data for public health action.

Introduction

Approximately 75% of the United States' population is connected to a public sewer system (1). However, sewer connectivity is not equally distributed in the US; western regions and urban areas tend to have higher rates of sewer connectivity (2). Wastewater monitoring, a crucial public health tool, utilizes existing wastewater infrastructure to collect a sample of untreated wastewater which is analyzed for human pathogens. Initially in response to the COVID-19 pandemic, wastewater monitoring programs were launched nationwide to track disease trends in the population within a sewershed, or the community area upstream of a wastewater sampling site (3). These data provide a community-level estimate of disease that is not impacted by an individual's access to healthcare or symptom severity. Throughout the pandemic, wastewater data served as an early indicator of increasing trends and became increasingly relied upon as at-home testing became commonplace and case-based surveillance became less reliable (4,5). Wastewater monitoring in the US has since expanded to include influenza viruses, including influenza A subtypes during an outbreak of avian influenza A (H5), respiratory syncytial virus (RSV), and other outbreak pathogens such as monkeypox virus, and measles virus (6,7). Public health officials analyze wastewater data alongside clinical surveillance data to gain a more comprehensive understanding of the diseases present in the community. Unlike clinical surveillance data, which can include patient demographic information for each sample, wastewater samples are not inherently linked to data describing the contributing population, even when sampling relatively closed populations like schools or healthcare facilities. As a result, information regarding the characteristics of groups or communities with higher or lower pathogen detections in wastewater, crucial for informing public health actions, is absent.

Although wastewater monitoring data does not innately include a description of the sample population, geographic data about the wastewater sampling site can be used to assess the attributes of populations monitored through wastewater. The Centers for Disease Control and Prevention's (CDC) National Wastewater Monitoring System (NWSS) collects different types of sample site location data including the county(ies) served and a sewershed polygon depicting the geographic area that contributes wastewater to that sampling site. Both county and sewershed geospatial data can be utilized to estimate

wastewater-monitored population characteristics. In 2022, when sewershed data submitted to NWSS were still largely incomplete, NWSS populations sampled were described in terms of the overall social vulnerability and urbanicity of the county(ies) served by the sampling sites (3). They observed that counties with NWSS testing were more urban and had slightly lower overall social vulnerability as compared to all US counties. A detailed evaluation of North Carolina's sewershed populations found that individual sewershed populations sometimes differed from county populations but that characteristics of wastewater-monitored sewershed populations aggregated to the state level were similar to statewide population characteristics (8).

According to the National Academies of Science Engineering and Medicine, an important characteristic of a national wastewater surveillance system is equitable distribution among population demographics (9,10). A better understanding of the sewershed community within wastewater monitoring networks can help to meet this objective. Sewershed population information also provides valuable context to wastewater data which will improve its interpretation and actionability. However, a lack of a national sewershed dataset has prevented the completion of a comprehensive geospatial analysis characterizing wastewater-monitored population representativeness. We leveraged sewershed geospatial data submitted to NWSS to investigate the wastewater-monitored population through two research questions: 1) Do sewershed populations monitored through CDC NWSS differ from the statewide populations with respect to certain demographic and social vulnerability characteristics, and if so, how do they differ and 2) have the differences changed as sampling sites were enrolled or dropped over time? This analysis highlights similarities and differences between monitored sewershed populations and broader populations and increases our understanding of how wastewater data can be used to support public health and public health action.

Methods

Data collection and pre-processing

Wastewater concentration data were submitted to CDC NWSS by state, tribal, local, and territorial (STLT) health departments, a CDC-managed contract for commercial testing, intended to supplement the geographic coverage of the STLT programs, and a private-academic partnership program. Sewershed geospatial data were submitted to CDC NWSS by STLT programs for their sites as well as commercial testing sites in their state. Based on the wastewater data received by NWSS from STLT and commercial testing sites as of November 2024, we generated a list of current sites, or those that had submitted sampling results during the most recent 3 months (August 2024 – October 2024). To investigate population changes over time, we created lists of sites at eight different time points (January and August of the years 2021, 2022, 2023, and 2024) to reflect sites sampling during peak and off-peak respiratory virus season. For each list of sampling sites, we joined the tabular data to georeferenced sewershed polygons, keeping the sewersheds that matched with a sampling site on the list. State boundary geospatial data was downloaded from the 2020 US Census (11). A state was included in the analysis if greater than 80 percent of their ELC funded wastewater treatment plant (WWTP) sampling sites or greater than 80 percent of their sampled population had a corresponding sewershed polygon. This activity was reviewed by CDC and was conducted consistent with applicable federal law and CDC policy.

Variables describing race, ethnicity, and group quarters (populations living or staying in a group living arrangement often with specific services or care provided) were obtained from the 2020 United States Census Redistricting Data, which were available at the census block level (12). We also analyzed variables from the 2018-2022 American Community Survey (ACS) that captured information about age, health insurance status, educational level, wealth, English proficiency, housing, unemployment, and disability status, all of which were available at the census tract level. American Community Survey 2018-2022 data at the tract level were obtained from ESRI's Living Atlas and compiled in ArcGIS Pro (13). To calculate the total population, we downloaded the Environmental Protection Agency's EnviroAtlas Dasymetric Allocation of Population, 2020 raster data for the continental United States, Alaska, and Hawaii (14). Lastly, we used a shapefile of nationwide Census tracts with information on the

CDC/ATSDR Social Vulnerability Index 2022 (SVI) (15). SVI analyzes variables from US census data to assess and rank 16 social factors such as poverty, crowded housing, race, and disability, to determine the social vulnerability of each Census tract or county across the United States. In addition to the social vulnerability percentile ranks for overall social vulnerability, the index also includes vulnerability related to four themes, including socioeconomic status (SES), household characteristics, racial and ethnic minority status, and housing type and transportation (SI Table 1). We analyzed overall SVI, theme-level SVI, and individual Census and ACS variables to understand whether populations differed with respect to a particular type of social vulnerability, or overall, and whether a given variable was driving vulnerability.

Geospatial analysis

We calculated the total population, the proportion of the population in each Census variable, and the average social vulnerability ranking for each polygon dataset of interest which included states, counties, the state's aggregated sewersheds, and individual sewersheds. To produce a multi-part polygon representing the state's aggregated sewershed, we merged the individual monitored sewershed polygons by state. This allowed us to analyze the statewide sewershed population without double-counting populations when sewersheds shared a boundary. We calculated the total population in sewersheds from the EnviroAtlas dasymetric population raster data and sewershed polygons. The EnviroAtlas data allocates population counts from US Census data to inhabitable areas as a 30-meter raster. These characteristics make the data highly suitable for estimating the population in a sewershed, which often has an irregular shape that can cross multiple administrative boundaries, such as counties, zip codes, and census tracts or blocks. To analyze the 2020 US Census and ACS demographic variables, we selected Census blocks or tracts that intersected each polygon of interest and weighted the total population according to the area of the block or tract that was inside the sewershed polygon. Then, the selected tracts or blocks were merged and summary statistics were calculated including the sum of the population and the average median household income. Population counts were converted to percentages by dividing by the total population using total population in blocks or tracts where appropriate. We also calculated the

population-weighted social vulnerability ranking of populations based on SVI 2022 tract level data for overall SVI and SVI related to the four themes. All geospatial analyses were conducted using Python (pandas, geopandas, rasterio, rasterstats, and scipy packages) (16–20).

To evaluate how the sewershed population compared to the statewide population, we calculated the difference between the aggregated sewershed population and the statewide population for each variable by calculating the percentage point difference for categorical variables and the percent difference for continuous variables. Testing for statistically significant differences was not used because the data were not independent (the populations in the monitored sewersheds were part of the state population) and did not follow a normal distribution. Therefore, we denoted potentially meaningful differences as +/-5 percent as previously described (8).

In a sub-analysis of the first research question, we assessed representativeness at a finer geographic scale to explore whether the characteristics of highly populated sewersheds were masking the characteristics of less populated sewersheds when sewersheds aggregated to the state level were compared to the state. We analyzed individual sewersheds and counties in a subset of states, Colorado, New York, North Carolina, and Wisconsin, which had complete sewershed polygon data and represented different regions of the US. We calculated the proportion of the population having certain demographic and social vulnerability characteristics in each monitored sewershed (sampling August - October 2024) and each county in the state. Then, a two-sample Kolmogorov-Smirnov test (KS-test) was performed for each variable to compare the distribution of the values between sewersheds and counties within each state. Distributions were considered significantly different when the KS test p-value was less than 0.05. Kernel density estimate (KDE) plots were used to visualize the distribution of observations in the sewersheds and counties by variable. For variables with significantly different distributions, we visually compared the sewershed population distribution relative to the county population distribution to determine the direction of the difference.

To learn whether representativeness has changed as sampling sites were added or removed over time, we compared the aggregated sewershed population and the statewide population at eight different time points (January and August, 2021-2024). We did this for a subset of states, New York, Wisconsin, Colorado, and North Carolina, which were early adopters of wastewater monitoring, had complete sewershed polygon data for sites sampling between 2021 and 2024, and represented different regions of the US. Using the geospatial data and methods already described, we analyzed the demographics and SVI of the state's aggregated sewershed population at each time point. We then compared the sewershed population to the state population by calculating the difference between the proportion of the sewershed population and the state population for each variable.

Results

Thirty-two states met the site or population coverage criteria and were included in the analyses (Table 1). The number of monitored sewersheds per state was highest in New York and lowest in Vermont (188 and 2, respectively), with a median of 21 monitored sewersheds per state. Utah included the highest percentage of the statewide population in monitored sewersheds (86%) while Vermont had the lowest statewide population coverage (1%). The median percentage of the state population included in wastewater monitored sewersheds was 42%.

Table 1. Characteristics of states analyzed.

State name	State population (2020) [a]	Monitored sewershed population (2020) [b]	% State population in monitored sewershed	County no.	Sewershed no.	Site no.
Arizona	7151502	3002238	41.98	15	14	15
California	39538223	16410707	41.51	58	49	54
Colorado	5773714	3115113	53.95	64	20	20
Delaware	989948	628020	63.44	3	6	12
Georgia	10711908	1918949	17.91	159	14	18
Hawaii	1455271	971777	66.78	5	9	9
Indiana	6785528	2182469	32.16	92	26	26
Kansas	2937880	538353	18.32	105	9	9
Kentucky	4505836	1548348	34.36	120	22	22
Maine	1362359	202499	14.86	16	19	21
Maryland	6177224	450056	7.29	23	9	9
Minnesota	5706494	3147757	55.16	87	27	35
Missouri	6154913	3021042	49.08	114	62	64
Nebraska	1961504	1300156	66.28	93	16	16
New Hampshire	1377529	266735	19.36	10	14	14
New Jersey	9288994	4605440	49.58	21	21	22
New Mexico	2117522	929094	43.88	33	9	11
New York	20201249	15557790	77.01	62	188	189
North Carolina	10439388	2900139	27.78	100	35	36
Ohio	11799448	5626205	47.68	88	75	75
Oklahoma	3959353	1588676	40.12	77	18	18
Oregon	4237256	2415094	57.00	36	31	37
Pennsylvania	13002700	2061718	15.86	67	33	38
Rhode Island	1097379	567678	51.73	5	11	12
South Carolina	5118425	1224836	23.93	46	23	24
Utah	3271616	2824508	86.33	29	35	35
Vermont	643077	8646	1.34	14	2	2
Virginia	8631393	4397442	50.95	95	31	38
Washington	7705281	4026997	52.26	39	30	30
West Virginia	1793716	318544	17.80	55	24	27
Wisconsin	5893718	2770710	47.01	72	42	43
Wyoming	576851	169027	29.30	23	7	7

A table showing 32 states analyzed including the state population, the wastewater-monitored population, and the numbers of monitored counties, sewersheds, and wastewater sites in the state sampling between August and October 2024.

[a] State populations were obtained from the 2020 US Census data.

[b] Sewershed populations were estimated from the EPA Dasymetric Allocation of Population US, 2020.

Comparing characteristics of aggregated sewershed and statewide populations

To address our first question, we compared the aggregated sewershed population within a state to the statewide population for 26 variables obtained from US Census, ACS, and SVI data. Across all states, the current monitored sewershed and statewide populations were similar (less than +/-5% difference) for 11/26 variables (less than 5 years, 65 years or older, no health insurance, disability, below federal poverty level, unemployed, native Hawaiian/Pacific Islander, limited English speaking, and group quarters - correctional, nursing, and military; Fig 1). Across 31 states there were four additional variables that had a less than +/-5% difference (less than 18 years, limited English speaking, Asian, and American Indian and Alaska Native; Fig 1). We found meaningful differences (greater than +/-5%) between the sewershed and state populations in multiple states for 7/26 variables (White alone, African American alone, Hispanic, median household income, bachelor's degree or higher, racial and ethnic minority status SVI, and housing type and transportation SVI; Fig 1). In general, the monitored sewershed populations had a lower proportion of Whites and a higher proportion of either African American, Hispanic, or Asian persons compared to the statewide population (Fig 1). Also, monitored sewershed populations tended to have higher educational attainment (Fig 2c) and lower median household income (Fig 2d). Meaningful differences in individual race and ethnicity characteristics were reflected as meaningfully higher racial and ethnic minority vulnerability in the monitored sewershed population (Fig 1 and Fig 2b). We also observed that overall social vulnerability and vulnerability related to each theme were often meaningfully different between the sewershed and the state population, and that the direction of the difference (higher or lower in the sewershed) often varied across states (Fig 1 and Fig 2a).

Fig 1. Differences between statewide monitored sewershed populations and the statewide populations. A heatmap showing the percentage differences between the aggregated statewide monitored

sewershed population proportion and the statewide population proportion for sites sampling August to October 2024. Shades of red indicate the proportion of the population is higher in the sewershed and shades of blue indicate the proportion of the population is lower in the sewershed. An * indicates that the difference is greater than +/- 5%. State abbreviations are displayed along the x-axis in alphabetical order.

Fig 2. Differences between statewide monitored sewershed populations and statewide populations. Maps showing the percentage differences between the aggregated statewide monitored sewershed population proportion (sites sampling August to October 2024) and the statewide population proportion for: a) housing type and transportation SVI, b) racial and ethnic minority status SVI, c) educational attainment, and d) median household income. Maps a, b, and c share a common legend.

However, in some states, the difference between the monitored sewershed and statewide population characteristics deviated from the patterns observed in most states. In Maryland, the monitored sewershed population had a meaningfully higher proportion of Whites, a lower proportion of minorities, and lower social vulnerability overall and across all themes which was opposite to what was observed in other states (Fig 1). The pattern was also different in Kansas, where we found that the sewershed and statewide populations were similar in race and ethnicity and that the sewershed population had meaningfully lower social vulnerability rankings (except for racial and ethnic minority status vulnerability) compared to the statewide population. A higher proportion of people living in group quarters, specifically colleges, was identified only in Vermont and New Hampshire’s monitored sewershed populations (Fig 1). Finally, some states’ wastewater monitored populations represented the statewide population well for measured variables. We observed that five states, Colorado, Nebraska, Oregon, South Carolina, and Utah, did not have any variables where the monitored sewershed and the statewide populations differed by greater than +/-5% (excluding median household income, which was meaningfully lower in the sewershed across all states; Fig 1). A complete list of differences between the sewershed and statewide population for each state and variable analyzed can be found in the supplemental material (SI Table 2).

Comparing population characteristics across individual sewersheds and counties

In the sub-analysis, the relationship between the distributions of populations in monitored sewersheds and populations in counties differed across the four states analyzed. In North Carolina, a comparison of populations in 35 monitored sewersheds with populations in the state's 100 counties showed that the sewershed distribution had significantly different and lower proportions of people 65 years or older, Whites, and disabled people, and higher proportions of Hispanics, limited English speakers, and those with a bachelor's degree. North Carolina's sewershed population distribution also had significantly different and lower median household income and lower vulnerability related to socioeconomic status, household characteristics, and racial and ethnic minority status than the population distribution in counties (Fig 3). In New York, populations across 188 monitored sewersheds had lower proportions of people with no health insurance, lower median household income and lower housing type and transportation SVI, and racial and ethnic minority status SVI compared to populations in New York's 62 counties. Only vulnerability related to household characteristics was meaningfully shifted towards higher values in New York's sewershed populations (Fig 3). In Colorado, population distributions were similar between the state's 20 monitored sewersheds and its 64 counties, except for median household income and racial and ethnic minority status vulnerability, where the sewershed populations included lower values (Fig 3). Wisconsin's 47 sewershed populations had lower proportions of people 65 years or older and lower median household income compared to populations in its 72 counties. In Wisconsin, the population distribution in monitored sewersheds was shifted towards higher values for overall SVI as well as vulnerability related to SES, household characteristics, and housing type and transportation (Fig 3). The KS statistic and associated p-value for each variable by state can be found in the supplemental material (SI Table 3).

Fig 3. Distributions of sewershed populations and county populations. Plots showing the distribution of the proportion of the population for select variables in each sewershed sampling August to October 2024 (orange) and each county (blue) within the state: A) 65 years or older, B) white, C) African American, D) Hispanic, E) limited English speaking, F) disability, G) without health insurance, H) bachelor's degree or higher, I) median household income, J) SES SVI, K) household characteristics SVI, L) racial and ethnic minority SVI, M) housing type and transportation SVI. An asterisk indicates a statistically significant difference (KS statistic p-value <0.05) between the sewershed and county distributions.

Comparing characteristics of the aggregated sewershed and statewide populations over time

For the second research question, we explored wastewater-monitored population representativeness over time (January and August, 2021-2024) in four states. In New York, the differences between the wastewater-monitored population and the statewide population have been in the same direction (higher or lower) but have decreased in magnitude as New York increased population coverage by more than tripling the number of monitoring sites between 2022 and 2024. Specifically, the wastewater monitored population in New York has consistently had a higher proportion of African Americans, Asians, and Hispanics and a lower proportion of Whites, and more residents with limited English-speaking ability, and higher social vulnerabilities than the statewide population (Fig 4c). In North Carolina, the monitored sewershed population consistently had a meaningfully different, higher proportion of African Americans, a lower proportion of Whites, and a higher proportion of people with a bachelor's degree, regardless of changes in the number of sampling sites or population coverage. While the direction of differences in North Carolina is consistent over time, the differences decreased when North Carolina had more sites and increased population coverage between January 2023 and January 2024 (Fig 4b). In contrast to findings in New York and North Carolina, Colorado's sewershed population

characteristics have been comparable to the statewide population characteristics over time, even though Colorado's wastewater program expanded to 63 sites in 2023 and then reduced its site count to 20 by August 2024 (Fig 4a). Finally, in Wisconsin, the direction and magnitude of the differences between the sewershed and statewide population characteristics were stable over time, even though the number of monitored sewersheds decreased from 70 to 47 between 2021 and 2024 (Fig 4d).

Fig 4. **Differences between statewide monitored sewershed populations and statewide populations and changes in total population and total sites sampled over time.** Heatmaps showing the percentage point differences between the proportion of the population monitored statewide sewersheds and the state and line graphs showing the total sewershed population and number of sewershed sites sampling in January or August of the years 2021, 2022, 2023, and 2024 in a) Colorado, b) North Carolina, c) New York, and d) Wisconsin. Dates on the x-axis are aligned to allow for comparisons between plots. An * in the heatmap indicates that the difference was greater than +/-5%.

Discussion

In this study, we leveraged sewershed polygon data to determine the characteristics of populations included in CDC NWSS and assess whether wastewater-monitored population characteristics are representative of broader populations. Our findings highlight the importance of having accurate data about the sewershed population and how under- or over-representation of certain groups can impact the interpretation of wastewater data for public health. In some states, including Colorado, Utah, and South Carolina, wastewater was sampled from sewersheds where the population represented the state population well, indicating that wastewater data can effectively serve as a proxy for disease trends across the state. In other states, we identified differences between the monitored sewershed and the state populations. In general, there was a higher proportion of racial and ethnic minorities and a lower proportion of Whites in sewershed populations. Also, the sewershed population had higher educational attainment, but at the same time, lower median household income and somewhat higher proportions of people living below the

federal poverty level compared to the state population. This suggests that although higher education is often linked with higher income, sewershed populations may experience greater income inequity, with a concentration of both high-income earners and low-income earners resulting in a lower median income (21,22). Factors such as socioeconomic status, income, race, ethnicity, and education, are important determinants of health because they shape individuals' access to resources which then influences their risk for poor health outcomes (23,24). Including a higher proportion of groups with greater risk factors and a higher burden of disease in wastewater monitoring is beneficial to public health as this can fill critical surveillance gaps and improve public health information about people who may experience a disproportionate burden of disease (25–27). However, wastewater data should be interpreted in consideration of these similarities and differences because there is a risk that viral levels detected in wastewater could be exaggerated when populations at higher risk of increased disease burden are over-sampled. By recognizing the similarities and differences between sewershed and broader populations, wastewater monitoring data can be effectively integrated with other clinical surveillance sources to inform public health decisions.

When we compared the aggregated sewershed population to the state population, we found that high population coverage did not always correspond with minor differences between the sewershed and the statewide population. Wastewater monitoring programs in Utah, Delaware, Nebraska, Hawaii, and New York included the majority of the state’s population, but characteristics of the wastewater-monitored population and the state population differed for some variables. In three states, New York, Delaware, and Hawaii, we observed meaningful differences in race, ethnicity and social vulnerability characteristics even though the wastewater-monitored population included 77%, 63%, and 67% of the state’s population, respectively. Sewer connectivity has been linked with certain population demographics and so, in some states, differences between the sewershed and state population characteristics may persist even when a large portion of the population is represented in wastewater monitoring (2). Conversely, Colorado and South Carolina included a smaller proportion of the state in wastewater monitoring, 28% and 24%

respectively, but attributes of the sewershed population closely matched the statewide population across all variables analyzed. These findings suggest that, in these states, although the wastewater-monitored population was relatively small, it reflected the overall diversity of the statewide population.

Since wastewater monitoring largely occurs in communities served by municipal sewer systems in highly populated, urban counties (3), comparing aggregated sewershed population characteristics to statewide population characteristics may have masked smaller sewersheds' population characteristics. For example, the distribution of the proportion of the population 65 years or older in individual sewersheds was significantly different and lower than the distribution in counties in the four states analyzed even though we did not find meaningful differences between the aggregated sewershed and the state population in 32 states. Understanding whether the elderly are over or under-represented in a state’s wastewater data is particularly important to public health because the elderly are at greater risk of severe disease or death due to respiratory illnesses (28). Additionally, we found that the proportion of the population with a bachelor’s degree or higher in sewersheds compared to counties was significantly different only in North Carolina and Wisconsin even though educational attainment was found to be higher in the sewershed population compared to the state population in the majority of states and meaningfully higher in six states. In North Carolina, we found several more differences not observed when comparisons were made at the state level, indicating that the less populous sewershed characteristics differed from populous sewershed characteristics or simply that the ratio of monitored sewersheds compared to counties (35 sewersheds vs 100 counties) necessitated a more granular analysis method. Considering these results and previous research that demonstrated differences between sewershed and county population characteristics (8), states might benefit from assessing population representativeness at different geographic levels. The differences between the distribution of populations in sewersheds and counties also demonstrate the importance of performing epidemiological studies based on the sewershed population rather than the county population when sub-county data are available.

In most states, sampling site composition has fluctuated over time, first expanding to cover a high proportion of the population during the height of the COVID-19 pandemic and then contracting as states scaled back their number of testing sites and added testing for additional pathogens. Increasing the percentage of the state's population included in wastewater monitoring over time did not greatly affect the differences between the wastewater monitored and the statewide population in New York, North Carolina, Wisconsin, and Colorado. Although substantially increasing wastewater monitoring coverage in New York improved wastewater representativeness, differences between the sewershed and state populations remained. Furthermore, changes in the number of sampling sites and population coverage between 2021 and 2024 in North Carolina, Colorado, and Wisconsin had minimal effect on representativeness. This implies that sites which were added or removed were relatively small or had similar population characteristics to the state.

This study was subject to several limitations. First, our analysis may not have included the entire wastewater-monitored population within each state. We assessed sewershed populations using sewershed polygon data submitted to CDC NWSS for ELC-funded sites, which may not include the entirety of wastewater monitoring sites within a state. Also, we included states in our analysis that had submitted a sewershed for at least 80% of their current wastewater monitoring sites or wastewater monitored population, to have sufficient data to estimate wastewater monitored population characteristics while including a majority of states. The analysis could be re-run when complete sewershed geospatial data are available. Second, using a spatial intersect may include people residing outside of the sewershed if a sewershed partially overlaps a block or tract. We attempted to account for this by area-weighting the US Census and ACS population counts and population-weighting the SVI ranks. Third, the KS test flagged differences between sewershed and county distributions as significant for some variables where the proportions of the population were very small, which complicates the interpretation of these results. Finally, the scope of this analysis was limited in several ways. Since sewershed data were only available for wastewater monitoring sites (past and present), we could not assess how characteristics of monitored

sewersheds compared to the overall sewered population or how the overall sewered population compared to the state population. Also, we did not attempt to account for the fact that sewershed populations are not static. People who do not reside in a sewershed may travel into a sewershed for school, work, or leisure, or vis-versa, and some sewersheds will experience more population fluctuation than others.

Future Directions

This study provides a framework for assessing wastewater-monitored population characteristics and comparing them with the broader population. Collecting a sewershed polygon for every wastewater sampling site submitting data to NWSS is an ongoing effort, and when complete, this analysis can be repeated to evaluate population representativeness for all states and at the national scale. The geospatial methods described here can be easily adapted to analyze additional population descriptions from US Census or other relevant data or to determine representativeness at different geographic scales, such as state-specific public health regions. Additionally, the method could be expanded to look beyond sewershed population characteristics to include assessing the presence of travel hubs, healthcare facilities, schools, or other points of interest in the sewershed. Future work can incorporate information about sewershed characteristics or representativeness into site selection tools so that wastewater data can efficiently and effectively meet public health needs. For example, when monitoring common respiratory illnesses such as COVID-19 or influenza, wastewater programs could prioritize sampling from sites that closely reflect the broader population to improve the relevance and utility of the data. (29). Alternatively, they might focus sampling efforts on sewersheds with high proportions of people over 65 years to monitor populations at higher risk for more severe illness, or sewersheds with high numbers of nursing home facilities and hospitals to track hospital-associated infections (30).

Conclusion

A wastewater sample represents a composite of the people in the sewershed regardless of individuals' demographics, socioeconomic conditions, or access to healthcare. This is the first study to analyze sewershed polygons for wastewater sites participating in CDC NWSS to assess how the wastewater-monitored population compared to broader populations. Through this research, we gained new perspectives about whether certain populations were over- or under-represented in a state's wastewater data and how sewershed population representativeness has changed as states enrolled and dropped sampling sites since NWSS was established in 2021. A better understanding of the sewershed population can provide insight for interpreting pathogens detected in wastewater, thereby strengthening the utility of wastewater monitoring as a tool for protecting the public's health.

Disclaimer

The findings and conclusions in this report are those of the authors and do not necessarily represent the official position of the Centers for Disease Control and Prevention.

Acknowledgements

We thank all state, tribal, local, and territorial health departments, wastewater utilities, and laboratories that have participated in the National Wastewater Surveillance System. Specifically, we thank Arizona Department of Health Services; California Department of Public Health and the many California wastewater utilities participating in the CDC NWSS program; Colorado Department of Public Health and Environment and participating Colorado wastewater utilities; Delaware Public Health; Georgia Department of Public Health; Hawaii State Department of Health, State Laboratories Division, Di, Doris, Yoong, Wen, and Steadmon, Maria; Indiana Department of Health; Kansas Department of Health and Environment; Kentucky Department for Public Health; Maryland Department of the Environment; Maine Department of Health and Human Services; Minnesota Department of Health, Zach Zirnhelt,

Epidemiologist Intermediate at MDH Austin Bell, (former) Senior Epidemiologist at MDH; Missouri Department of Health; North Carolina Wastewater Monitoring Network at the North Carolina Department of Health and Human Services; Nebraska Department of Health and Human Services, Derry Stover and Likhitha Duggirala; New Hampshire Department of Health and Human Services; The New Jersey Department of Health's (NJDOH) Communicable Disease Service (CDS) and Public Health Environmental Laboratories (PHEL); New Mexico Department of Health Wastewater Surveillance Program and participating New Mexico municipalities; New York State Department of Health; Ohio Department of Health; Oregon Health Authority Public Health Division and Oregon State University; Pennsylvania Department of Health; Rhode Island Department of Health; South Carolina Department of Public Health (SC DPH); Utah Department of Health and Human Services, Utah Wastewater Surveillance System; Virginia Department of Health; Vermont Department of Health; Washington state Department of Health; Wisconsin Wastewater Monitoring Program, Wisconsin Department of Health Services, Wisconsin State Laboratory of Hygiene, University of Wisconsin-Milwaukee; WV Bureau of Public Health, and WaTCH-WV (Wastewater Testing for Community Health in WV) partners WVU, Marshall University, and participating WWTP operators and WWTP lab personnel; Wyoming Department of Health

References

1. US EPA O of W. About Small Wastewater Systems [Internet]. 2015 [cited 2025 Aug 24]. Available from: https://www.epa.gov/small-and-rural-wastewater-systems/about-small-wastewater-systems

2. Yu Q, Olesen SW, Duvallet C, Grad YH. Assessment of sewer connectivity in the United States and its implications for equity in wastewater-based epidemiology. PLOS Glob Public Health. 2024 Apr 17;4(4):e0003039.

3. Adams C, Bias M, Welsh RM, Webb J, Reese H, Delgado S, et al. The National Wastewater Surveillance System (NWSS): From inception to widespread coverage, 2020–2022, United States. Sci Total Environ. 2024 May;924:171566.

4. Olesen SW, Imakaev M, Duvallet C. Making waves: Defining the lead time of wastewater-based epidemiology for COVID-19. Water Res. 2021 Sep 1;202:117433.

5. Keshaviah A, Huff I, Hu XC, Guidry V, Christensen A, Berkowitz S, et al. Separating signal from noise in wastewater data: An algorithm to identify community-level COVID-19 surges in real time. Proc Natl Acad Sci U S A. 2023 Jul 25;120(31):e2216021120.

6. Boehm AB, Hughes B, Duong D, Chan-Herur V, Buchman A, Wolfe MK, et al. Wastewater concentrations of human influenza, metapneumovirus, parainfluenza, respiratory syncytial virus, rhinovirus, and seasonal coronavirus nucleic-acids during the COVID-19 pandemic: a surveillance study. Lancet Microbe. 2023 May 1;4(5):e340–8.

7. Chen W, Bibby K. Temporal, spatial, and methodological considerations in evaluating the viability of measles wastewater surveillance. Sci Total Environ. 2025 Jan 10;959:178141.

8. Reckling SK, Hu XC, Keshaviah A. Equity in wastewater monitoring: Differences in the demographics and social vulnerability of sewered and unsewered populations across North Carolina. PloS One. 2024;19(10):e0311516.

9. National Academies of Sciences E and Medicine. Wastewater-based Disease Surveillance for Public Health Action [Internet]. Washington, DC: The National Academies Press; 2023. Available from: https://nap.nationalacademies.org/catalog/26767/wastewater-based-disease-surveillance-for-public-health-action

10. National Academies of Sciences E and Medicine. Increasing the Utility of Wastewater-based Disease Surveillance for Public Health Action: A Phase 2 Report [Internet]. Washington, DC: The National Academies Press; 2024. Available from: https://nap.nationalacademies.org/catalog/27516/increasing-the-utility-of-wastewater-based-disease-surveillance-for-public-health-action

11. U.S. Census Bureau. Cartographic Boundary Files, States 1:500,000 (national) [Internet]. 2023 [cited 2024 Aug 17]. Available from: https://www.census.gov/geographies/mapping-files/time-series/geo/cartographic-boundary.html

12. Esri. USA Census 2020 Redistricting Blocks [Internet]. 2021 [cited 2024 Jul 17]. Available from: https://www.arcgis.com/home/item.html?id=b3642e91b49548f5af772394b0537681

13. Esri. ArcGIS Pro v3.1. Redlands, CA; 2023.

14. United States Environmental Protection Agency. Dasymetric Allocation of Population U.S., 2020 v.1.1 [Internet]. 2020 [cited 2024 Aug 5]. Available from: https://www.epa.gov/enviroatlas/data-download?token=SCNavq_vQNrdFWrVq7g5fZ7BV5FACDWSqOAAzaZRLW0

15. Centers for Disease Control and Prevention/ Agency for Toxic Substances and Disease Registry/ Geospatial Research, Analysis, and Services Program. CDC/ATSDR Social Vulnerability Index 2022 Database U.S. [Internet]. 2022 [cited 2024 Nov 15]. Available from: https://www.atsdr.cdc.gov/placeandhealth/svi/data_documentation_download.html

16. The pandas development team. pandas-dev/pandas: Pandas [Internet]. Zenodo; 2020. Available from: https://doi.org/10.5281/zenodo.3509134

17. Jordahl K et al. geopandas [Internet]. Zenodo; 2024. Available from: https://zenodo.org/records/3946761#.Xy24LC2ZPOQ

18. Virtanen P, Gommers R, Oliphant TE, Haberland M, Reddy T, Cournapeau D, et al. SciPy 1.0: fundamental algorithms for scientific computing in Python. Nat Methods. 2020 Mar;17(3):261–72.

19. Gillies S. Rasterio: Access to geospatial raster data [Internet]. Mapbox; 2024. Available from: https://github.com/mapbox/rasterio

20. Perry M. rasterstats [Internet]. 2024. Available from: https://pypi.org/project/rasterstats/

21. National Center for Education Statistics. Annual Earnings by Educational Attainment. Condition of Education. U.S. Department of Education, Institute of Education Sciences [Internet]. 2024 [cited 2025 Aug 17]. Available from: https://nces.ed.gov/programs/coe/indicator/cba/annual-earnings

22. Thiede BC, Butler JLW, Brown DL, Jensen L. Income Inequality across the Rural-Urban Continuum in the United States, 1970–2016. Rural Sociol. 2020;85(4):899–937.

23. Whitman A, Lew ND, Chappel A, Aysola V, Zuckerman R, Sommers BD. Addressing Social Determinants of Health: Examples of Successful Evidence-Based Strategies and Current Federal Efforts. 2022;

24. Town M. Racial and Ethnic Differences in Social Determinants of Health and Health-Related Social Needs Among Adults — Behavioral Risk Factor Surveillance System, United States, 2022. MMWR Morb Mortal Wkly Rep. 2024;73.

25. Graham G. Disparities in Cardiovascular Disease Risk in the United States. Curr Cardiol Rev. 2015 Aug;11(3):238–45.

26. Gray DM, Anyane-Yeboa A, Balzora S, Issaka RB, May FP. COVID-19 and the other pandemic: populations made vulnerable by systemic inequity. Nat Rev Gastroenterol Hepatol. 2020 Sep;17(9):520–2.

27. Tai DBG, Sia IG, Doubeni CA, Wieland ML. Disproportionate Impact of COVID-19 on Racial and Ethnic Minority Groups in the United States: a 2021 Update. J Racial Ethn Health Disparities. 2022 Dec 1;9(6):2334–9.

28. Häder A, Köse-Vogel N, Schulz L, Mlynska L, Hornung F, Hagel S, et al. Respiratory Infections in the Aging Lung: Implications for Diagnosis, Therapy, and Prevention. Aging Dis. 2023 Jul 31;14(4):1091–104.

29. Muralidharan A, Olson R, Bess CW, Bischel HN. Equity-centered adaptive sampling in sub-sewershed wastewater surveillance using census data. Environ Sci Water Res Technol. 2025;11(1):136–51.

30. Zulli A, Chan EMG, Shelden B, Duong D, Xu XRS, White BJ, et al. Prospective study of Candida auris nucleic acids in wastewater solids in 190 wastewater treatment plants in the United States suggests widespread occurrence. mBio. 2024 Jul 23;15(8):e00908-24.

Supporting information

SI Table 1. Variables included in the analysis. Variables are listed with the variable name, source, and geographic level of the data.

SI Table 2. The percentage point difference between the proportion of the population in the aggregated sewershed (sites sampling August to October 2024) and the proportion of the population in the state.

SI Table 3. Results of the two-sample Kolmogorov-Smirnov test. A KS test compared the distribution of the proportions of the population with certain demographic and social vulnerability characteristics in individual sewersheds and counties within a state for sites sampling August to October 2024. A * denotes that the proportion of the population in group quarters (all types) were very small or zero and so, KS test results should be considered unreliable.

Figure 1

Click here to access/download;Figure;Figure1.tif

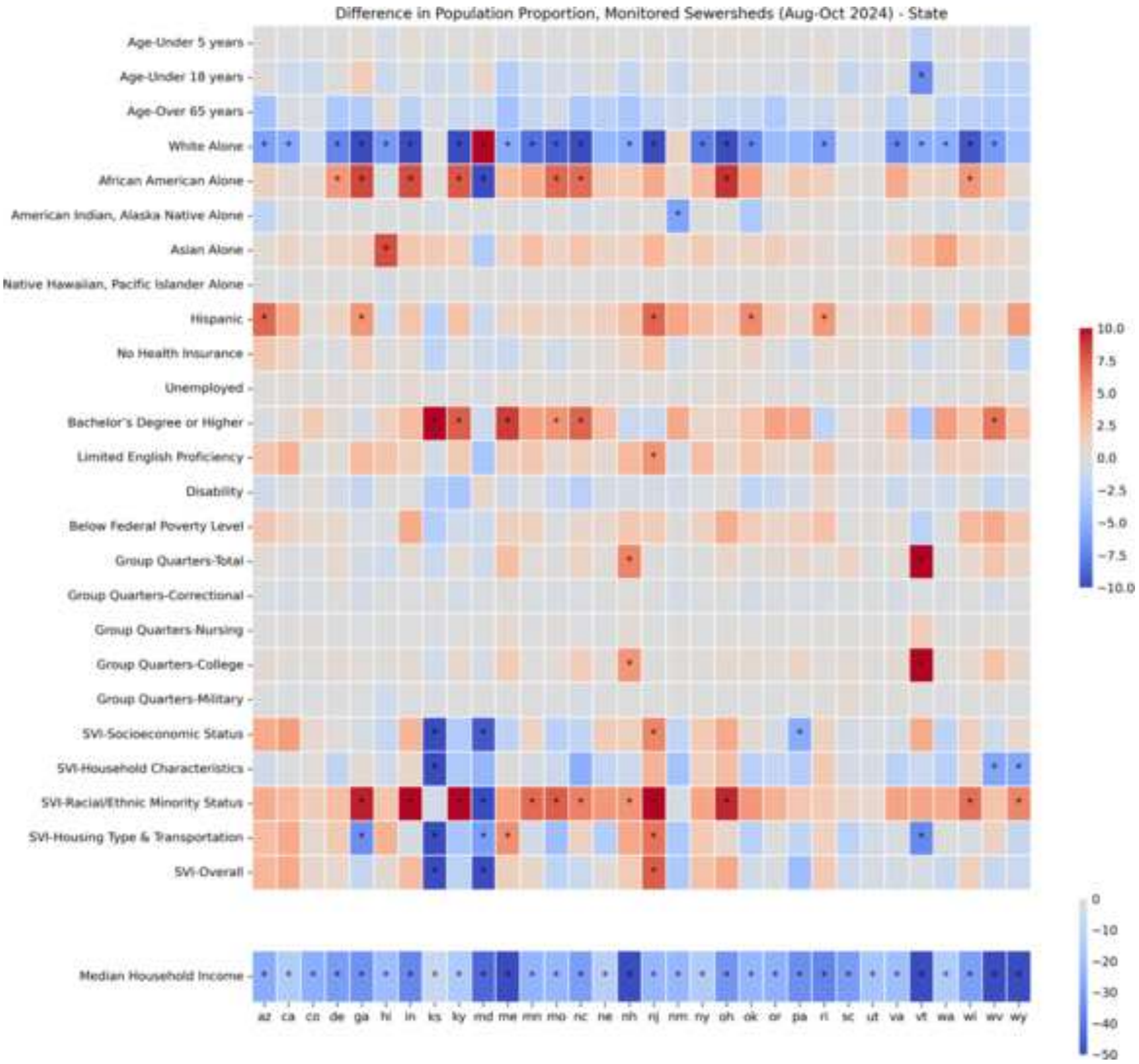

Figure 2

Click here to access/download;Figure;Figure2.tif

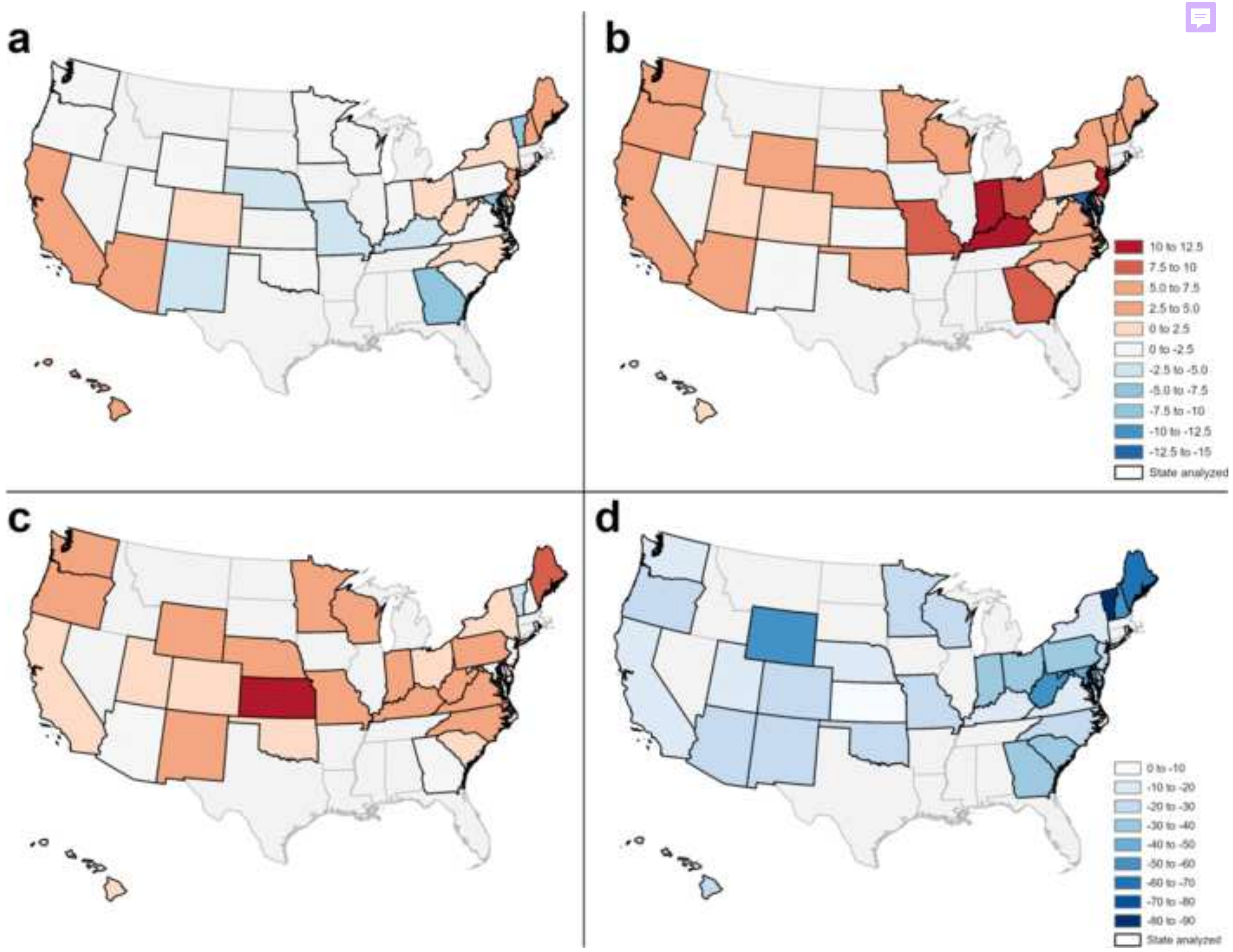

Figure 3

Click here to access/download;Figure;Figure3.tif

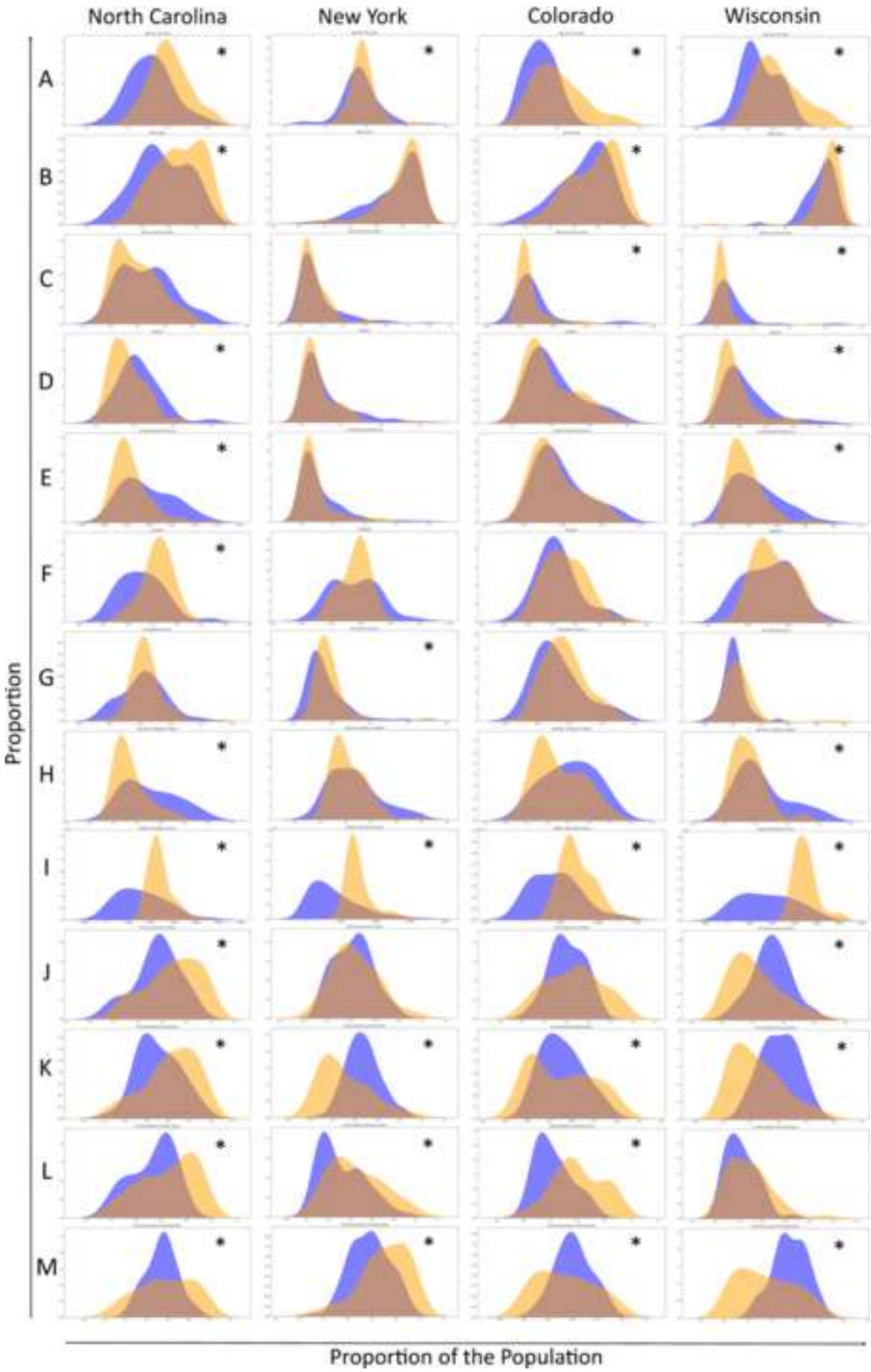

Figure 4

Click here to access/download;Figure;Figure4.tif

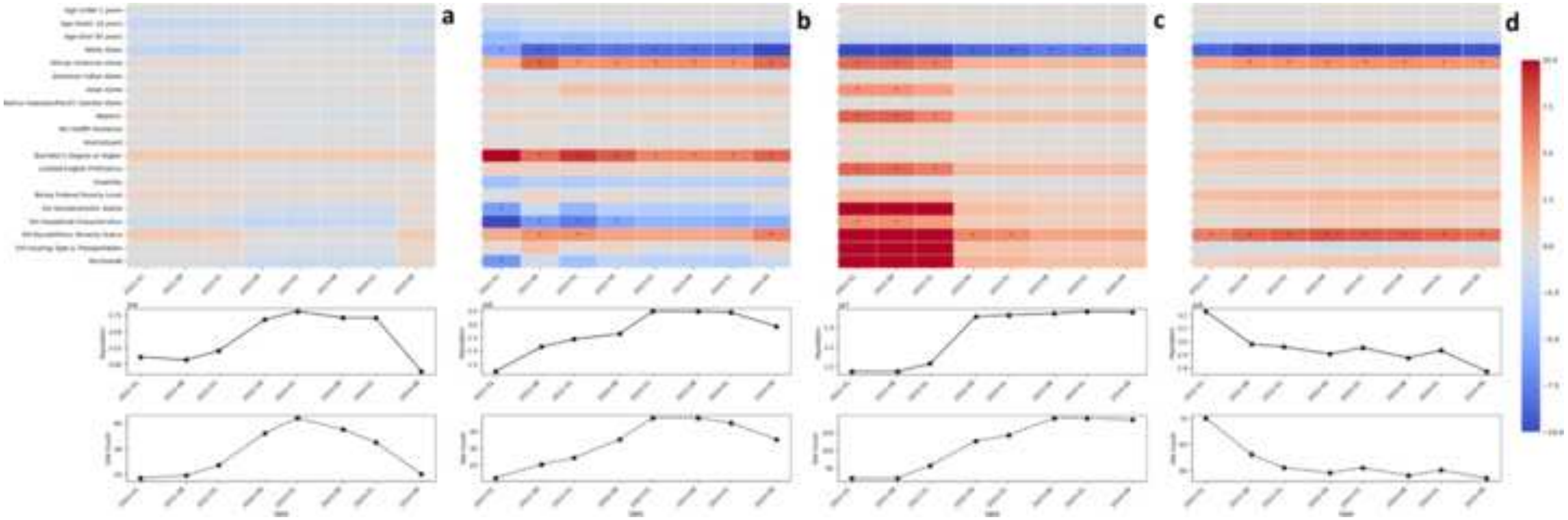

SI Table 1

Click here to access/download
Supporting Information
SITable1.xlsx

SI Table 2

Click here to access/download
Supporting Information
SITable2.xlsx

SI Table 3

Click here to access/download
Supporting Information
SITable3.xlsx
