# Supplementary material for: A geospatial analysis comparing wastewater-monitored sewershed and statewide populations for 32 states participating in CDC’s National Wastewater Surveillance System, 2021-2024
Source: PLOS Glob Public Health. 2026 Apr 22;6(4):e0006243. doi: 10.1371/journal.pgph.0006243 (PMC13102245; doi:10.1371/journal.pgph.0006243)
Supplement: Attachment — Submitted filename: PGPH-D-25-03983-comments.docx [file pgph.0006243.s007.docx]

This manuscript presents a carefully designed and technically sophisticated geospatial analysis of wastewater-monitored populations across multiple spatial and temporal scales. The use of sewershed polygons, dasymetric population estimation, and population-weighted social vulnerability metrics is methodologically sound. However, several aspects of the statistical framework used to quantify and interpret population differences warrant further clarification and, in some cases, reconsideration. These issues primarily concern the choice of effect metrics, the reporting of underlying quantities, and the consistency of inferential approaches.

**1. Interpretation of proportional differences based solely on the absolute scale**

The analysis of representativeness is based on absolute differences in proportions (percentage point differences), with a fixed ±5% threshold used to identify potentially meaningful discrepancies between aggregated sewershed populations and statewide populations. While absolute differences are intuitive and easy to communicate, their interpretability depends strongly on baseline proportion. For example, an absolute difference of 5 percentage points can correspond to a several-fold relative change for rare characteristics, but only a modest proportional change for highly prevalent characteristics.

From a statistical perspective, relying exclusively on absolute differences may therefore obscure heterogeneity in effect magnitude across variables and lead to differential interpretation depending on scale. This concern is particularly relevant for characteristics with low baseline proportions (e.g., certain racial or group quarters categories), where relative measures such as relative risk (RR) may better capture substantive change. The authors may consider incorporating relative effect measures as complementary summaries, or at a minimum explicitly acknowledge the scale dependence of absolute differences and their implications for interpretation.

**2. Reporting of effect metrics without corresponding baseline proportions**

Relatedly, the manuscript focuses on reporting differences in proportions without systematically presenting the underlying proportions for the statewide and aggregated sewershed populations. From a statistical reporting standpoint, effect sizes are most interpretable when accompanied by the baseline quantities from which they are derived. Without access to the original proportions, the reader cannot assess the relative magnitude of differences, evaluate potential scale effects, or compute alternative metrics such as relative risk.

Providing the underlying proportions—at least in supplementary materials—would substantially improve transparency and reproducibility, and would allow readers to independently assess the robustness of the conclusions to alternative effect metrics. Including these values would not require reanalysis of the data, but would materially strengthen the interpretability of the results.

**3. Consistency of inferential strategy across analytical components**

The manuscript employs different inferential strategies across analyses. For comparisons between aggregated sewershed populations and statewide populations, the authors avoid formal hypothesis testing due to non-independence and normality assumptions, opting instead for a heuristic ±5% threshold. In contrast, for n the comparison of distributions between individual sewersheds and counties, the authors apply the Kolmogorov–Smirnov test, a nonparametric test that does not rely on normality assumptions.

While both approaches are defensible in isolation, the rationale for applying a formal nonparametric test in one context but not considering analogous rank-based or distributional methods in the aggregated comparison would benefit from further clarification. Even acknowledging partial dependence between populations, some nonparametric or resampling-based approaches, such as the bootstrapping method, could be explored or at least discussed. Explicitly articulating why such methods were not pursued would improve methodological coherence.

In addition, the choice of ±5% as a cutoff for identifying “potentially meaningful” differences merits further justification. It is unclear whether this threshold is grounded in substantive public health relevance, empirical precedent, or heuristic analogy to conventional statistical significance levels. A brief sensitivity discussion—addressing whether alternative thresholds (e.g., ±1% or ±10%) would materially alter the qualitative conclusions—would help readers assess the robustness of the findings.

My recommendation is for a major revision
