# Supplementary material for: A geospatial analysis comparing wastewater-monitored sewershed and statewide populations for 32 states participating in CDC’s National Wastewater Surveillance System, 2021-2024
Source: PLOS Glob Public Health. 2026 Apr 22;6(4):e0006243. doi: 10.1371/journal.pgph.0006243 (PMC13102245; doi:10.1371/journal.pgph.0006243)
Supplement: Attachment — Submitted filename: nwss_pop_response_to_reviewers_03_2026.docx [file pgph.0006243.s010.docx]

Dear Editor and Reviewers,

Thank you for allowing us to submit a revised draft of the manuscript “A geospatial analysis comparing wastewater-monitored sewershed and statewide populations for 32 states participating in CDC’s National Wastewater Surveillance System, 2021-2024” for publication in the journal PLOS Global Public Health. We appreciate the time and effort you dedicated to providing feedback on our manuscript and are grateful for the insightful comments and valuable improvements to our paper. We have incorporated most of the suggestions made by the reviewers. Please see below where we have addressed the comments point-by-point (our replies are in red) and made changes to the manuscript.

**Journal Requirements:**

1. We have noticed that you have uploaded Supporting Information files, but you have not included a list of legends. Please add a full list of legends for your Supporting Information files after the references list.

Author’s Response: A full list of captions is included for the Supporting Information files after the references section.

2. We note that your Data Availability Statement is currently as follows: Links to publicly available databases used in this project are provided in the reference section. The data supporting the findings of this study are available in the Supplemental Information. Sewershed polygons are unavailable to access due to concerns about sensitive infrastructure information.
 
 Please confirm at this time whether or not your submission contains all raw data required to replicate the results of your study. Authors must share the “minimal data set” for their submission. PLOS defines the minimal data set to consist of the data required to replicate all study findings reported in the article, as well as related metadata and methods (<https://journals.plos.org/plosone/s/data-availability#loc-minimal-data-set-definition>).
 
 For example, authors should submit the following data:
 
 - The values behind the means, standard deviations and other measures reported;
 - The values used to build graphs;
 - The points extracted from images for analysis.
 
 Authors do not need to submit their entire data set if only a portion of the data was used in the reported study.
 
 If your submission does not contain these data, please either upload them as Supporting Information files or deposit them to a stable, public repository and provide us with the relevant URLs, DOIs, or accession numbers. For a list of recommended repositories, please see <https://journals.plos.org/plosone/s/recommended-repositories>.
 
 If there are ethical or legal restrictions on sharing a de-identified data set, please explain them in detail (e.g., data contain potentially sensitive information, data are owned by a third-party organization, etc.) and who has imposed them (e.g., an ethics committee). Please also provide contact information for a data access committee, ethics committee, or other institutional body to which data requests may be sent. If data are owned by a third party, please indicate how others may request data access.

Author’s response:

We have added several new files to the Supplemental Information including a table of the proportions of the state and sewershed populations (SI Table 4), a table of the sewershed to state population ratios (SI Table 5), and a processing workflow diagram to help readers understand the methods (SI Fig 1). Titles and captions for new tables and figures have been added. We have also edited the data availability statement to better explain the availability of the sewershed data.

*“Links to publicly available databases used in this project are provided in the reference section. The data supporting the findings of this study are available in Supplemental Information. Sewershed polygons are unavailable due to wastewater utilities’ ethical concerns about disclosing protected infrastructure information and can be requested directly from the wastewater utility.”*

***SI Fig 1.*** *Diagram of data analysis workflow. A diagram showing the data and methods including data collection, geospatial analysis, and calculation of various effect metrics.*

***SI Table 4. The proportion of the population for each variable in the aggregated sewershed (sites sampling August to October 2024) and statewide population.***

***SI Table 5. The ratios between the proportion of the population in the aggregated sewershed (sites sampling August to October 2024) and statewide population for each variable.***

3. Some material included in your submission may be copyrighted. According to PLOS’s copyright policy, authors who use figures or other material (e.g., graphics, clipart, maps) from another author or copyright holder must demonstrate or obtain permission to publish this material under the Creative Commons Attribution 4.0 International (CC BY 4.0) License used by PLOS journals. Please closely review the details of PLOS’s copyright requirements here: PLOS Licenses and Copyright. If you need to request permissions from a copyright holder, you may use PLOS's Copyright Content Permission form.

Please respond directly to this email or email the journal office and provide any known details concerning your material's license terms and permissions required for reuse, even if you have not yet obtained copyright permissions or are unsure of your material's copyright compatibility.

Potential Copyright Issues:

a. Figure 2: please (a) provide a direct link to the base layer of the map (i.e., the country or region border shape) and ensure this is also included in the figure legend; and (b) provide a link to the terms of use / license information for the base layer image or shapefile. We cannot publish proprietary or copyrighted maps (e.g. Google Maps, Mapquest) and the terms of use for your map base layer must be compatible with our CC-BY 4.0 license.

Note: if you created the map in a software program like R or ArcGIS, please locate and indicate the source of the basemap shapefile onto which data has been plotted.

If your map was obtained from a copyrighted source please amend the figure so that the base map used is from an openly available source. Alternatively, please provide explicit written permission from the copyright holder granting you the right to publish the material under our CC-BY 4.0 license.

Please note that the following CC BY licenses are compatible with PLOS license: CC BY 4.0, CC BY 2.0 and CC BY 3.0, meanwhile such licenses as CC BY-ND 3.0 and others are not compatible due to additional restrictions.

If you are unsure whether you can use a map or not, please do reach out and we will be able to help you. The following websites are good examples of where you can source open access or public domain maps:

* U.S. Geological Survey (USGS) - All maps are in the public domain. (<http://www.usgs.gov>) 
* PlaniGlobe - All maps are published under a Creative Commons license so please cite “PlaniGlobe, <http://www.planiglobe.com>, CC BY 2.0” in the image credit after the caption. (<http://www.planiglobe.com/?lang=enl>) 
* Natural Earth - All maps are public domain. (<http://www.naturalearthdata.com/about/terms-of-use/>)

If the reviewer comments include a recommendation to cite specific previously published works, please review and evaluate these publications to determine whether they are relevant and should be cited. There is no requirement to cite these works unless the editor has indicated otherwise. 

Please review your reference list to ensure that it is complete and correct. If you have cited papers that have been retracted, please include the rationale for doing so in the manuscript text, or remove these references and replace them with relevant current references. Any changes to the reference list should be mentioned in the rebuttal letter that accompanies your revised manuscript. If you need to cite a retracted article, indicate the article’s retracted status in the References list and also include a citation and full reference for the retraction notice.

Author’s response:

Thank you for this reminder. We have checked that all references are complete and correct. The maps in Figure 2 show publicly available state boundary data. We have added a link to the data in the Figure 2 caption.

*“State boundaries are publicly available from https://www.census.gov/geographies/mapping-files/time-series/geo/cartographic-boundary.html.”*

**Editor Comments:**

**Point 1.** Abstract: The abstract would benefit from the inclusion of a few plain-language summary statements, such as: “Currently, NWSS covers X% of the U.S. population. In X of Y states, NWSS provides good representation of the statewide population; however, in Z states, NWSS may over- or underrepresent certain populations based on factors such as race, income, or other demographics.”

Author’s response:

Thank you for your suggestion. We re-organized the abstract and added the population coverage of CDC NWSS during the study period and a high-level summary (lines 39-43). Some of the more detailed information was omitted so that the abstract remained under the 300-word limit.

*“In Fall of 2024, CDC NWSS included sites in all 50 states covering 41% of the US population. In 5 of 32 states analyzed the wastewater-monitored population represented the statewide population across all variables except median household income. In 27 states, we observed that wastewater-monitored populations may over- or underrepresent certain populations based on factors like race and ethnicity, education, or social vulnerability.”*

**Point 2.** Line 49: Citation #1 is 10 years old. A more appropriate reference would be the 2025 WHO/UNICEF JMP, which includes the proportion of households connected to sewer systems in the United States.

Author’s response:

Thank you for sharing an updated reference! We have revised the percentage of the US population that is connected to municipal wastewater based on the information in the report and substituted the new reference for the older reference in line 49.

*“Approximately 84% of the United States’ population is connected to a public sewer system (1).”*

**Point 3.** Lines 63–67: This section is generally not factually accurate. Most publications on WBE over the last five years explicitly include contributing population size in the methods section. This also typically includes population demographics such as income level, race, or ethnicity. These lines should be removed.

Lines 68–70: In a piped wastewater system in the United States, the number of contributing users is always known, as this information is required under EPA wastewater treatment plant permit requirements. These lines should be removed.

Author’s response:

Thank you for your suggestions. We have removed lines 63-67. We have also reworded the beginning of the next paragraph (lines 64-70) to clarify that wastewater sample metadata submitted to CDC’s National Wastewater Surveillance System do not include population demographic information but that this information can be generated by using the geographic data that is submitted for each site.

*“Wastewater represents a composite sample of the community upstream of a collection site. Although metadata submitted to the Centers for Disease Control and Prevention’s National Wastewater Surveillance System (CDC NWSS) does not include detailed population demographic information, the accompanying geographic metadata can be used to infer characteristics of the wastewater-monitored population. CDC NWSS collects different types of sample site location data including the county(ies) served and a sewershed polygon representing the geographic area that contributes wastewater to that sampling site.”*

**Point 4.** Table 1 is unclear and needs to be reframed. Using Kentucky as an example: throughout most of the pandemic, there were only five wastewater treatment plants reporting to NWSS (serving a population of less than 1 million). However, Table 1 also includes data through late 2024, when additional ELC-funded sites were added. Reframing the table on a year-by-year basis would better illustrate that some states have experienced decreases in the percentage of population covered, while others (such as Kentucky) have seen increases. As a result, analyses based on late 2024 data differ substantially from those based on 2021 sample sites in terms of population representation. As currently presented, the state population (2020) does not align with the monitored sewershed population (2020) when the 2024 number of sewersheds is applied. For Kentucky, the monitored sewershed population (2020) should be approximately 1,000,000, not 1,548,348 as shown in the table. One option would be to update the table to use 2024 population estimates. This same issue applies to lines 274–301.

Author’s response:

We apologize that Table 1 was unclear and hope that our revisions to the Table 1 headers, title, and caption will clarify the information provided in the table. We meant for Table 1 to summarize the wastewater-monitored population coverage and sites included in the analysis that compared the wastewater-monitored population to the statewide population. We used the EPA’s dasymetric population raster, which was derived from US Census 2020 block population, to accurately estimate the sewershed population. Therefore, we used Census 2020 data to estimate the statewide population so the populations (sewershed and state) were calculated from the same year. We have updated the Table 1 title (line 192) to clarify that the table shows wastewater monitoring coverage and the Table 1 caption (lines 194-199) to explain that 2020 population data was used to calculate the percent of the statewide population.

*“****Table 1. Wastewater-monitoring coverage in 32 states analyzed.****”*

*“A table showing information about the percentage of the state population included in wastewater monitoring, counties with wastewater monitoring, monitored sewersheds, and monitored wastewater sites sampling (August to October 2024) for 32 states analyzed. A state was included in the analysis if it had a sewershed for at least 80% of its monitoring sites or monitored population. Sewershed and state populations were estimated from US Census 2020 data to ensure that the percentage of the statewide population included in wastewater monitoring was calculated from the same year’s data.”*

**Review Comments to the Author**

**Reviewer #1:** This manuscript presents a carefully designed and technically sophisticated geospatial analysis of wastewater-monitored populations across multiple spatial and temporal scales. The use of sewershed polygons, dasymetric population estimation, and population-weighted social vulnerability metrics is methodologically sound. However, several aspects of the statistical framework used to quantify and interpret population differences warrant further clarification and, in some cases, reconsideration. These issues primarily concern the choice of effect metrics, the reporting of underlying quantities, and the consistency of inferential approaches.

**Point 1.** Interpretation of proportional differences based solely on the absolute scale
The analysis of representativeness is based on absolute differences in proportions (percentage point differences), with a fixed ±5% threshold used to identify potentially meaningful discrepancies between aggregated sewershed populations and statewide populations. While absolute differences are intuitive and easy to communicate, their interpretability depends strongly on baseline proportion. For example, an absolute difference of 5 percentage points can correspond to a several-fold relative change for rare characteristics, but only a modest proportional change for highly prevalent characteristics.
From a statistical perspective, relying exclusively on absolute differences may therefore obscure heterogeneity in effect magnitude across variables and lead to differential interpretation depending on scale. This concern is particularly relevant for characteristics with low baseline proportions (e.g., certain racial or group quarters categories), where relative measures such as relative risk (RR) may better capture substantive change. The authors may consider incorporating relative effect measures as complementary summaries, or at a minimum explicitly acknowledge the scale dependence of absolute differences and their implications for interpretation.

Author’s response:

Thank you for the detailed and constructive suggestions to improve the quality of our manuscript. We agree that adding an additional effect metric can better illustrate differences for variables where the proportion of the population is small. In line with your suggestion, we have added an assessment of the ratio of the sewershed proportion of the population to the statewide proportion of the population. We have added additional text about this analysis to the methods (lines 156-159).

*“In addition to the absolute difference, we calculated the ratio of the sewershed population proportion to the statewide population proportion to highlight differences for variables with small populations such as the proportion living in group quarters or unemployed.”*

We have also added additional results based on the ratio metric:

*“The ratio between the sewershed and statewide population further demostrated that Asians were over-represented in wastewater and that sewershed populations included a higher proportion of limited English speakers.” (lines 211-213)*

*“The ratio between income in the sewershed and income in the state better exemplified the income gap; the sewershed populations’ income was 25% lower in half of states analyzed and 50% lower in 5 states.” (lines 214-216)*

*“Finally, based on the population ratio, we also saw differences in the proportion of the population in group quarters where generally, sewersheds included a lower percentage of people living in correctional group quarters and a higher percentage of people living in college group quarters.” (lines 221-224)*

**Point 2.** Reporting of effect metrics without corresponding baseline proportions
Relatedly, the manuscript focuses on reporting differences in proportions without systematically presenting the underlying proportions for the statewide and aggregated sewershed populations. From a statistical reporting standpoint, effect sizes are most interpretable when accompanied by the baseline quantities from which they are derived. Without access to the original proportions, the reader cannot assess the relative magnitude of differences, evaluate potential scale effects, or compute alternative metrics such as relative risk.

Providing the underlying proportions—at least in supplementary materials—would substantially improve transparency and reproducibility, and would allow readers to independently assess the robustness of the conclusions to alternative effect metrics. Including these values would not require reanalysis of the data, but would materially strengthen the interpretability of the results.

Author’s response:

Thank you for pointing this out. We agree that providing the underlying proportions for the aggregated sewershed and statewide populations will improve the transparency and reproducibility of our analysis and helps meet the ‘minimal data set’ requirement. We have added the sewershed and state proportions for each variable in a new table in the Supplemental Information (SI Table 4).

***SI Table 4. The proportion of the population for each variable in the aggregated sewershed (sites sampling August to October 2024) and statewide population.***

**Point 3.** Consistency of inferential strategy across analytical components
The manuscript employs different inferential strategies across analyses. For comparisons between aggregated sewershed populations and statewide populations, the authors avoid formal hypothesis testing due to non-independence and normality assumptions, opting instead for a heuristic ±5% threshold. In contrast, for n the comparison of distributions between individual sewersheds and counties, the authors apply the Kolmogorov–Smirnov test, a nonparametric test that does not rely on normality assumptions.

While both approaches are defensible in isolation, the rationale for applying a formal nonparametric test in one context but not considering analogous rank-based or distributional methods in the aggregated comparison would benefit from further clarification. Even acknowledging partial dependence between populations, some nonparametric or resampling-based approaches, such as the bootstrapping method, could be explored or at least discussed. Explicitly articulating why such methods were not pursued would improve methodological coherence.

Author’s response:

We thank the reviewer for their insight and appreciate the opportunity to clarify our approach. Our choice of analytical methods was driven by the distinct nature of the data underlying the two different comparisons. When comparing the aggregated sewershed and the statewide populations, only two values were compared. Therefore, we didn’t feel that rank-based tests and bootstrapping approaches would be appropriate since these are designed to work with a collection of data points. However, when comparing the distribution of sewershed values to county values, we felt that the two sample KS test was appropriate because it did not assume normality and could account for both differences in the center and the shape of the two distributions. We have added an additional sentence to explain the benefits of the KS test in the methods (lines 171-174).

*“The KS-test, which is sensitive to differences in both the center and the shape of the distributions, produced a KS statistic, indicative of the maximum difference between the distributions, and an associated p-value.”*

**Point 4.** In addition, the choice of ±5% as a cutoff for identifying “potentially meaningful” differences merits further justification. It is unclear whether this threshold is grounded in substantive public health relevance, empirical precedent, or heuristic analogy to conventional statistical significance levels. A brief sensitivity discussion—addressing whether alternative thresholds (e.g., ±1% or ±10%) would materially alter the qualitative conclusions—would help readers assess the robustness of the findings.

Author’s response:

We agree with the reviewer that our justification for using +/-5% was unclear. Per the reviewer’s suggestion, we have performed a sensitivity assessment of several difference thresholds (+/-1%, 3%, 5%, 7%, and 10%) to identify a threshold that denoted ‘meaningful differences’ while ignoring inconsequential differences or ‘noise’. After conducting the sensitivity analysis, we found our initial choice of a +/-5% difference remained the best choice. A short discussion of the sensitivity of different thresholds has been added to the methods (lines 153-156).

*“We assessed the sensitivity of various difference thresholds (plus or minus 1%, 3%, 5%, 7% and 10%) to identify the threshold that could capture potentially meaningful differences. We chose +/-5% because across most variables, it balanced the need to capture subtle yet real distinctions while minimizing noise from minor variations and aligned with previous similar analyses (8).”*

We also added text to the limitations paragraph in the discussion to highlight that the choice of threshold could change the interpretation of the results (lines 403-406).

*“Third, using a +/-5% difference threshold affected the number of variables deemed meaningfully different. Choosing a lower threshold would reveal more differences in smaller populations, but it also may detect minor variations that lack practical importance in larger populations.”*

**Reviewer #2:** Overall impressions: This research assesses selected population demographics of wastewater monitoring sewersheds in selected US states in terms of their efficacy in representing the greater populations of which they are a part - either the county or state level. A secondary research question assesses the temporal stability of the relationships between sewershed demographics and state demographics. Through the GIS-based methodology, which apportions sewersheds with demographic data through small area population estimates, the authors elucidate the high variable nature of the relationships between sewershed demographics and those of the larger spatial units they are nested within. The compelling argument that this paper makes to me is that as a general practice, based on the current distribution of NWSS monitoring sites we should not be using wastewater monitoring data to make claims about public health situations in areas beyond the the boundaries of the sewersheds they are sampling. This is very important, as data such as wastewater monitoring data may be used to draw conclusions that those data do not support. The manuscript would be strengthened by addressing this more directly and citing instances where this fallacy occurs. The research also supports the notion that sewersheds can be designed to capture specific populations that may be more or less representative of larger spatial units. Understanding the population demographics or a prospective sewershed prior to monitoring could yield better outcomes and lower costs.

General Feedback: The paper is well organized and provides good background and context on wastewater monitoring and the NWSS from which the spatial data were drawn.

**Point 1.** Could the significance of this research be strengthened through other arguments? Does wastewater monitoring within specific sewersheds claim to be representative of larger units (counties, states)? Do your results speak to the need for more representative sampling site selection? This paper is really interesting, but I think you could make a stronger case for why it is important. (comment in the marked-up PDF)

Author’s response:

We thank the reviewer for pointing out additional ways that our findings could be relevant. We recognize that states have different goals for their wastewater monitoring programs and that representativeness is just one way of prioritizing sampling sites We have added a sentence to describe when representativeness may be of higher or lower importance in the first paragraph of the Discussion (lines 322-325). Also, in the future directions section of the Discussion, we describe how achieving a representative group of sampling sites could be included in site prioritization efforts (lines 424-431).

*“Our findings suggest that wastewater monitoring can sometimes capture representative populations, which is useful when tracking widespread respiratory pathogens like SARS-CoV-2 or influenza, while in other cases, a deliberate over-sampling of at-risk groups may be more desirable.”*

**Point 2.** I recommend revising the methods section for clarity, as it is quite dense and laborious right now. I think some tables and figures could greatly aid the reader in understanding the data and processing workflows, which could then facilitate condensing the body text. I have provided specific comments in the marked up PDF file.

Author’s response:

We appreciate the reviewer’s thoughtful comments provided in the marked-up PDF and this specific feedback about the density of the methods section. We have addressed many of the reviewer’s comments from the PDF throughout the manuscript. Also, we have added a workflow diagram figure to the Supplemental Information (SI Figure 1) to show the analysis process. We have also added additional references for the table of variables analyzed (SI Table 1) in lines 116 and 119 of the tracked changes document and condensed some of the language in the methods section to make it more concise.

“***SI Fig 1. Diagram of data analysis workflow.*** *A diagram showing the data and methods including data collection, geospatial analysis, and calculation of various effect metrics.*” (lines 561-562)

**Point 3.** On the quantitative side, the use of the arbitrary “meaningful difference” threshold of +/-5% is of concern. If there are existing usages of this threshold in the literature it would be helpful to cite them. Perhaps there are other ways to evaluate the significance of these differences?

Author’s response:

Thank you. We agree with the reviewer that the reason for denoting potentially meaningful differences as +/-5% was unclear and that an additional metric would help identify differences when the proportion of the population is relatively small. To address these concerns, we looked at different thresholds (+/-1%, 3%, 5%, 7%, and 10%) to see how the number of ‘meaningful differences’ changed at different thresholds (lines 153-156).

*“We assessed the sensitivity of various difference thresholds (plus or minus 1%, 3%, 5%, 7% and 10%) to identify the threshold that could capture potentially meaningful differences. We chose +/-5% because across most variables, it balanced the need to capture subtle yet real distinctions while minimizing noise from minor variations and aligned with previous similar analyses (8).”*

We also added an additional effect metric where we calculated the ratio between the proportion of the sewershed population and the proportion of the state population to capture differences in small populations such as those living in group quarters. We have revised the methods and the results accordingly.

*“In addition to the absolute difference, we calculated the ratio of the sewershed population proportion to the statewide population proportion to highlight differences for variables with small populations such as the proportion living in group quarters or unemployed.”* (lines 156-159).

**Point 4.** The figures are generally good, but please see my comment regarding the symbology on the maps. I recommend considering alternative data classifications with fewer classes.

Author’s response:

Thank you for this suggestion. For the maps in Figure 2, we have condensed the classes so that the different classes can be more easily distinguished and each class is represented on the maps. We have submitted a revised Figure 2 with the manuscript revisions.
